# In Situ EBSD Study of Deformation Behavior at Grain Scale of Inconel 718 Alloy During Tensile Test at 650 °C

**DOI:** 10.3390/ma18091934

**Published:** 2025-04-24

**Authors:** Lijun Sang, Junxia Lu, Xiaopeng Cheng, Yuefei Zhang, Ze Zhang

**Affiliations:** 1 College of Materials Science and Engineering, Beijing University of Technology, Beijing 100124, China; sanglijun@bigc.edu.cn (L.S.); junxialv@bjut.edu.cn (J.L.); 2 School of Materials Science and Engineering, Zhejiang University, Hangzhou 310058, China; zezhang@zju.edu.cn

**Keywords:** Inconel 718 alloy, tensile test, in situ EBSD, grain boundary, Schmid factor

## Abstract

In order to clarify the deformation mechanism of Inconel 718 (IN718) alloy at the grain scale during tensile deformation, the deformation behaviors of IN718 alloy were investigated at 650 °C using an in situ electron backscatter diffraction (EBSD) tensile testing method. The evolution of grain morphology, crystallographic orientation, activated slip systems, grain boundaries evolution, and strain-induced misorientation were systematically analyzed during the tensile test. The results showed that the grains were elongated along the tensile direction, and the grain boundaries also became significantly curved. Meanwhile, the EBSD studies illustrated that the changes in local misorientation within individual grains were non-uniform and generally began at the grain boundaries. The low-angle grain boundaries (LAGBs) were first formed near the high-angle grain boundaries (HAGBs) and gradually expanded into the interior of the grains. The activation of the slip system and the Schmid factor were characterized and calculated based on the slip traces on the deformed grain surface. The evolution of local strain within the grains was evidenced by a kernel average misorientation (KAM) map. Finally, the plastic deformation mechanism at the grain scale was discussed in detail based on our experimental results.

## 1. Introduction

Inconel 718 (IN718) alloy is one of the most common nickel-based superalloys and has been widely used for structural components operating at temperatures up to 650 °C, due to its excellent mechanical properties and good corrosion resistance at high temperatures [1,2,3]. IN718 alloy is simultaneously subjected to high temperature and high stress during long-term service, resulting in a continuous microstructural evolution in the alloy. The evolution of the microstructure directly deteriorates the performance of the materials [4,5]. Thus, understanding the microstructural features and their evolutionary behavior during deformation that affect the final performance at elevated temperatures is very crucial. For polycrystalline materials, the evolution of microstructure usually occurs at the grain scale [6]. It is well-known that the macroscopic deformation of materials is a process that accumulates from local damage and gradually expands to the global level [7,8]. Therefore, the local deformation within the individual grains will ultimately affect the macroscopic deformation of materials. However, due to the grain boundaries and different grain orientations, each grain will be in a different stress state, and even under uniaxial tensile loading, the deformation process is very complex. Therefore, it is necessary to study the local deformation of the individual grains to understand the macroscopic deformation mechanism of the alloy.

In recent years, many researchers have studied the microstructure deformation behavior of IN718 alloy at different temperatures to understand how deformation behavior and microstructure correlate with damage nucleation mechanisms [9,10,11,12]. Boehlert et al. [13] performed ambient-temperature tensile experiments on IN718 alloy to identify the active slip systems and the stress at which the slip systems were activated. Charpagne et al. [14] investigated the relation between the slip localization behavior and the 3D microstructure of IN718 during uniaxial tensile loading at room temperature (RT) and found strong correlations between the location of slip occurrence and specific microstructure configurations. Texier et al. [15] studied the irreversible deformation in relation to the microstructure for IN718 alloy from RT to 650 °C using high-resolution digital image correlation (HR-DIC) techniques, and revealed that strain localization at the grain boundaries is particularly intense and not necessarily assisted by slip in adjacent grains at 650 °C. Jullien et al. [16] investigated the grain size effects on the early plastic strain localization and slip transfer at the grain boundaries for IN718 alloy at 650 °C, and found that the fine-grain microstructures have extensive strain localization at grain boundaries, while the coarse-grain microstructure is more prone to intragranular slip development and slip localization near twin boundaries. Gao et al. [17] analyzed the effects of grain size on the tensile behavior of IN718 alloy at RT using an in situ scanning electron microscope (SEM) and EBSD techniques and revealed that the geometrically necessary dislocation (GND) density depended on the grain size and the orientation of individual grains. Chen et al. [18] investigated the effects of grain boundary misorientation on the plastic deformation of an IN718 alloy using an in situ tensile test at 650 °C in combination with the crystal plasticity finite-element method and clarified the mechanism of the influence of grain boundary misorientation on IN718 microplastic deformation. The above studies indicate that grain orientation and boundaries play a significant role during plastic deformation, particularly when the material is subjected to tensile stress.

In situ SEM tensile testing combined with EBSD is an efficient and effective technique for obtaining detailed microstructural features or misorientation parameters, such as grain boundaries, grain orientation, kernel average misorientation (KAM), and active slip systems during plastic deformation [19]. Moreover, a qualitative and quantitative analysis of these deformation structures can also be obtained through post-processing [20]. In our previous work, it has been found that the plastic deformation of IN718 alloy at 650 °C is mainly characterized by crystal slip [21]. However, only slip traces, slip band density, and changes in grain shape and morphology were observed through SEM. This cannot determine the relationship between the activation of slip systems and local strain, nor can it identify crystallographic information such as grain orientation and changes in grain boundary misorientation with strain. The variation in this crystallographic information is of great importance for understanding the deformation mechanism of IN718 alloy at the microscale.

In the present study, the in situ EBSD uniaxial tensile stage was used to investigate the evolution of microstructural features in IN718 alloy at 650 °C. This study aims to clarify the deformation mechanism of IN718 alloy at the grain scale during tensile deformation at service temperature by analyzing and discussing microstructural evolution, activation of slip systems, strain-induced misorientation, substructure formation, and grain boundary evolution.

## 2. Materials and Methods

### 2.1. Sample Preparation

A 30 mm-diameter hot-rolled bar of IN718 alloy (Central Iron and Steel Research Institute, Xueyuan South Road No. 76, Haidian District, Beijing, China) was used in this study. The nominal chemical composition (wt.%) of the alloy is shown in Table 1. The bar was first subjected to a heat treatment procedure consisting of a solution heat treatment at 1050 °C for 1 h, followed by water quenching. Then, the bar was aged at 725 °C for 15 h, followed by air quenching.

**Figure 1:**
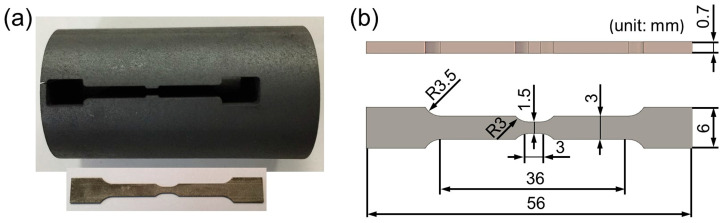
(**a**) In situ tensile sample; (**b**) dimensions of the in situ tensile sample.

For the in situ tensile test, the tensile samples were wire-cut into a dog-bone-shaped geometry with a gage length of 3 mm, a gage thickness of 0.7 mm, and a gage width of 1.5 mm, as shown in Figure 1, using electrical discharge machining. For the SEM observations, the samples were ground with SiC abrasive papers (300 to 2000#) and 0.5 µm diamond paste until mirror smooth and then etched with a mixture solution of 5 g CuCl_2_, 100 mL HCl, and 100 mL C_2_H_5_OH for 1 min at RT. Finally, to meet the surface requirement for EBSD, the samples were electrochemically polished in a solution of HClO_4_ and C_2_H_5_OH with a voltage of 18 V at RT for 15 s.

### 2.2. In Situ EBSD Tensile Test

**Figure 2:**
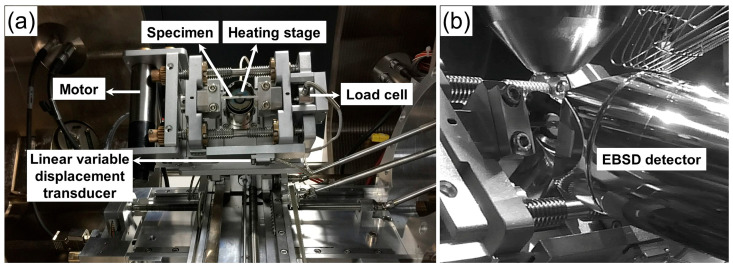
(**a**) Installation of in situ EBSD high-temperature tensile stage in SEM; (**b**) relative position of EBSD detector and tensile stage.

The EBSD tensile stage developed by the authors [22] was installed inside the SEM chamber (TESCAN S8000, TESCAN, Brno, Czech Republic), as shown in Figure 2a. The working status of the EBSD detector (Symmetry S1, Oxford Instruments, Oxford, UK) during the tensile test is shown in Figure 2b. In order for the EBSD detector to collect more backscattered electron signals and ensure higher pattern resolution, the sample and tensile stage were fixed at a pre-tilted angle of 70°. Before tensile loading, the tested sample was heated to the required temperature of 650 °C and held for 30 min to ensure temperature uniformity along the gage region. The temperature was measured by connecting a thermocouple to the lower face of the sample. The tensile test was conducted in displacement-controlled mode at a rate of 1 μm/s (comparable strain rate of 3.3 × 10^−4^/s). The tensile test could be paused at various deformation stages to record the SEM micrographs and EBSD data. During the pause stage, the constant tensile load was maintained through the locking mechanism of the motor. After data acquisition, the test was resumed by starting the motor and using displacement-controlled conditions. The tensile load–displacement curve was recorded in real time via the control system.

**Figure 3:**
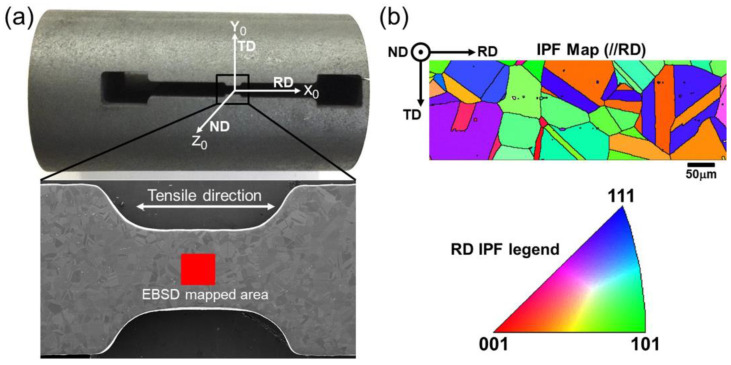
Schematic of the reference frame for data recording during EBSD scanning: (**a**) reference frame for SEM and EBSD; (**b**) reference frame of IPF map.

The EBSD test was conducted at an accelerating voltage of 20 kV, a beam current of 10 nA, and a step size of 0.5 µm. The EBSD patterns were collected at a frequency range of 600–800 Hz. The loading direction and frame of reference for the EBSD data analysis in this study are illustrated in Figure 3a. Figure 3b shows the reference frame for inverse pole figure (IPF) maps. All of the EBSD data and the related maps were post-processed using the HKL Channel 5 software (5.12.74.0, Oxford Instruments, Oxford, UK). The detailed process of determining the activated slip systems and calculating the Schmid factor (SF) can be referred to the Ref. [23].

## 3. Results

### 3.1. Morphology Changes During Tensile Deformation

**Figure 4:**
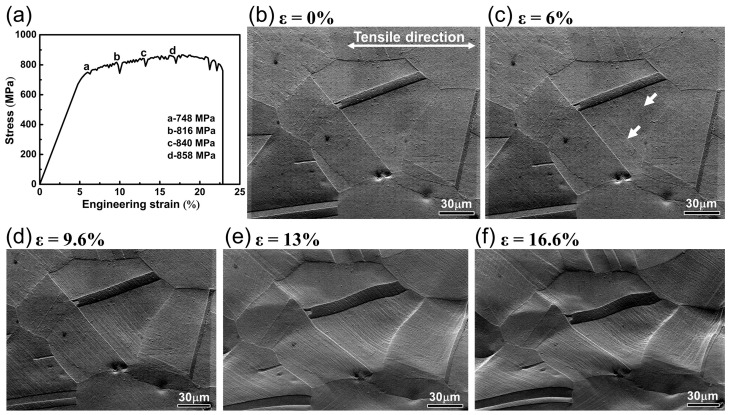
(**a**) Engineering stress–strain curve; (**b**–**f**) in-situ SEM images of morphology of the sample surface during in situ tensile test at 650 °C.

Figure 4a shows the engineering stress–strain curve of IN718 alloy during in situ EBSD tensile testing at 650 °C. During the tensile testing, EBSD scans were performed on the same deformation area by pausing the test at strains of 6%, 9.6%, 13%, and 16%, corresponding to points a, b, c, and d on the stress–strain curve. The time for each EBSD scan is approximately 30 min. Therefore, the slight stress drops in the curve result from stress relaxation during EBSD scanning. Figure 4b–f illustrate the changes in morphology of the sample surface under different strains. At a strain of 6%, compared to the undeformed microstructure, the sample surface remained flat without significant changes, but rare slip lines began to appear, as indicated by the white arrows in Figure 4c. When the strain increased to 9.6%, slip lines appeared inside multiple grains, and the density of the slip lines gradually increased, forming slip bands. The surfaces of the grains with dense slip lines began to become uneven. During the high strain levels, as shown in Figure 4e,f, the slip band density and the roughness of the grain surface both increased. The grains were elongated along the tensile direction, and the grain boundaries also became significantly curved.

### 3.2. Grain Orientation Evolution with Straining

**Figure 5:**
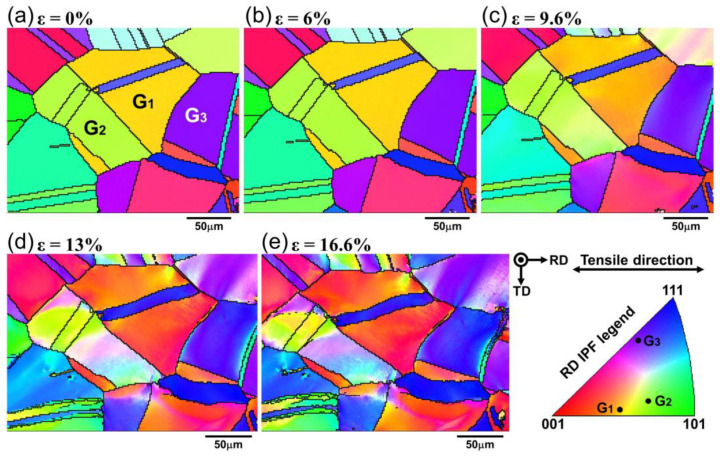
IPF maps of grain orientations parallel to the tensile direction at engineering strain of (**a**) 0%, (**b**) 6%, (**c**) 9.6%, (**d**) 13%, and (**e**) 16.6%. The exact crystallographic orientation of grains G_1_, G_2_, and G_3_ marked in (**a**) is given in the IPF legend.

Figure 5 shows the IPF maps at different strain levels. Crystallographic orientation changes were identifiable based on color variation within the grains, and crystallographic orientation can be roughly tracked from the colored IPF legend shown in Figure 5. It can be seen from Figure 5a that the grain orientations are random. However, the initial EBSD map does not indicate a preferred orientation for the surface grains in the scanned region. At a strain of 6%, the color inside a single grain remained uniform without significant changes, indicating that the grain orientation has not changed significantly. As the strain increased from 9.6% to 16.6%, it can be observed that there is a significant change in the grain orientation, in terms of the color of the individual grains, such as grain G_1_ changing from beige to red, and two different colors appearing inside grain G_2_. These indicate that the lattice gradient changed significantly within severely deformed grains, and the grain orientation rotated. In addition, it was found that these color changes started from the grain boundaries and gradually expanded into the interior of the grains, ultimately dividing the grain into different color regions.

### 3.3. Evolution of Grain Boundaries

**Figure 6:**
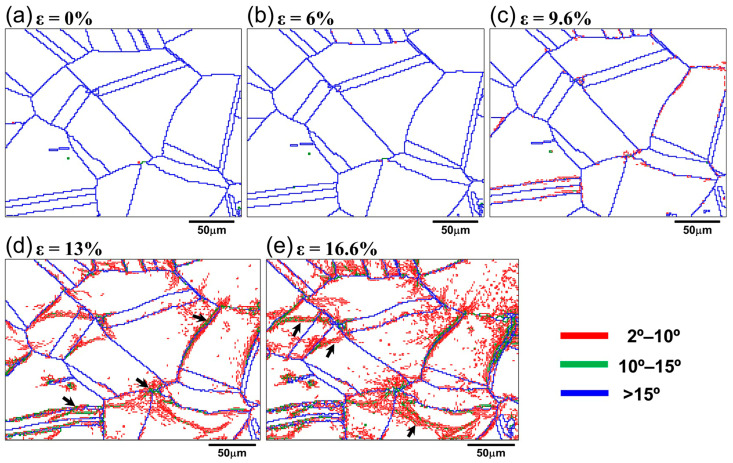
Grain boundary distribution maps at engineering strain of (**a**) 0%, (**b**) 6%, (**c**) 9.6%, (**d**) 13%, and (**e**) 16.6%.

Figure 6 shows grain boundary maps at different strains. In grain boundary maps, different colors represent grain boundaries at different angles. The grain boundaries with misorientation angles exceeding 15° and 2–15° were classified here as high-angle grain boundaries (HAGBs) and low-angle grain boundaries (LAGBs), respectively. As shown in Figure 6a,b, the grain boundaries were mainly HAGBs before deformation and at a low strain level. At a strain of 9.6%, the formation of the LAGBs smaller than 10° was observed near HAGBs, trident grain boundaries, and twin boundaries (TBs), as shown by the red scatter lines in Figure 6c. When the strain increased to 13%, LAGBs less than 10° significantly increased and began to expand from HAGBs to the interior of the grains. At the same time, it can be observed that green lines began to appear near some HAGBs, indicating the formation of LAGBs larger than 10°, as indicated by the black arrows in Figure 6d. As the strain increased to 16.6%, the density of the LAGBs (<10°) formed along the HAGBs also increased. The dispersed LAGBs within the grains began to aggregate and form wider structures, dividing the original grains into smaller sub-grain structures, as marked by the black arrows in Figure 6e.

**Figure 7:**
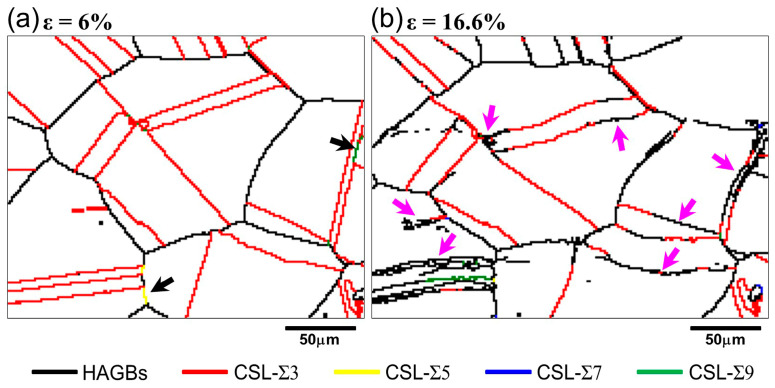
HAGBs and TBs distribution maps at engineering strain of (**a**) 6% and (**b**) 16.6%.

The distribution of coincidence site lattice (CSL) grain boundaries and HAGBs at strains of 6% and 16.6% is illustrated in Figure 7. It can be seen that CSL grain boundaries were mainly composed of the Σ3 TBs marked by red lines, and there were also a few Σ5 and Σ9 grain boundaries, as indicated by black arrows in Figure 7a. At a strain of 16.6%, as shown in Figure 7b, many Σ3 TBs transformed into HAGBs in segments, as marked by purple arrows. The integrity of the annealing twin was destroyed, and deformation twins were not observed.

In order to accurately analyze the evolution of grain boundaries during deformation, the changes in grain boundaries were quantitatively evaluated by the statistical variation of grain boundary misorientation. Here, the length percentage of different grain boundaries was used to quantify the relative density changes during deformation. This method may introduce errors in the measurement of the misorientation angles due to the severe deformation of grains at high strain levels, thereby affecting the quantification of grain boundary density. However, tracking the evolution of different grain boundaries by measuring their respective densities is the simplest and most straightforward way to describe the evolution of grain boundaries during deformation [24] because it can avoid some of the drawbacks of conventional methods, such as incomplete grain boundaries, in the determination of grain size in deformed samples.

**Figure 8:**
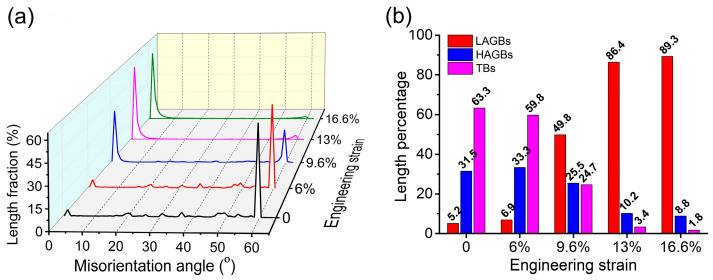
(**a**) Distribution of grain boundary misorientation with strain; (**b**) the quantitative comparison of LAGBs, HAGBs, and TBs at different engineering strain levels.

Figure 8a shows the distribution of the length fraction of the grain boundaries corresponding to misorientation angles between 0–65° at different strain levels. It can be seen that the fraction of TBs and HAGBs decreased continuously with strain, especially the TBs, which decreased more significantly, while the fraction of LAGBs continued to increase. Obviously, the strain of 9.6% is the turning point of this transformation. At a strain of 9.6%, as shown in Figure 8b, the relative density of the TBs decreased from 63.3% before deformation to 24.7%, while the LAGBs increased from 5.2% to 49.8%. When the strain increased to 16.6%, the density of the TBs decreased to 1.8%, while the LAGBs increased to 89.3%. The density of the HAGBs showed a slight increase at a strain of 6%, but no newly formed HAGBs are observed in Figure 6b. This indicated that the small increase in HAGBs was mainly due to the transformation of TBs.

### 3.4. Slip System Activity and Schmid Factor

**Figure 9:**
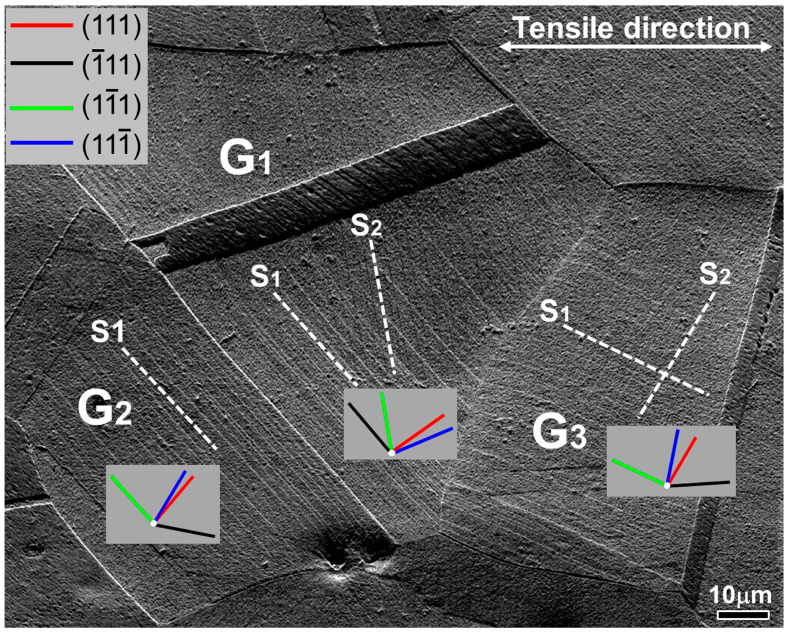
Determination of the slip planes corresponding to the slip traces on the sample surface at strain of 9.6%.

In order to better understand the crystal slip mechanism during plastic deformation, the activation of the slip system and the Schmid factor were characterized and calculated based on the slip traces on the deformed grain surface. At a strain of 9.6%, two sets of intersecting slip traces were observed on the surface of grains G_1_ and G_3_, as shown in Figure 9 and labeled as S_1_ and S_2_, respectively. This indicates that two sets of different slip systems were activated inside both grains, resulting in multiple slips. Only one set of slip traces appeared inside grain G_2_, indicating that only one slip system was activated under the resolved shear stress. Table 2 lists the determined slip planes within grains G_1_, G_2_, and G_3_, as well as the corresponding SF values for the three slip directions, and bolds the determined slip direction and SF value. It can be found that the SF values of the activated slip systems in grains G_1_ and G_2_ are both close to 0.5 (the maximum SF), indicating a soft orientation for the two grains. Conversely, grain G_3_ has a hard orientation due to its lower SF value (0.39–0.4), which makes it difficult to dislocation slip.

**Figure 10:**
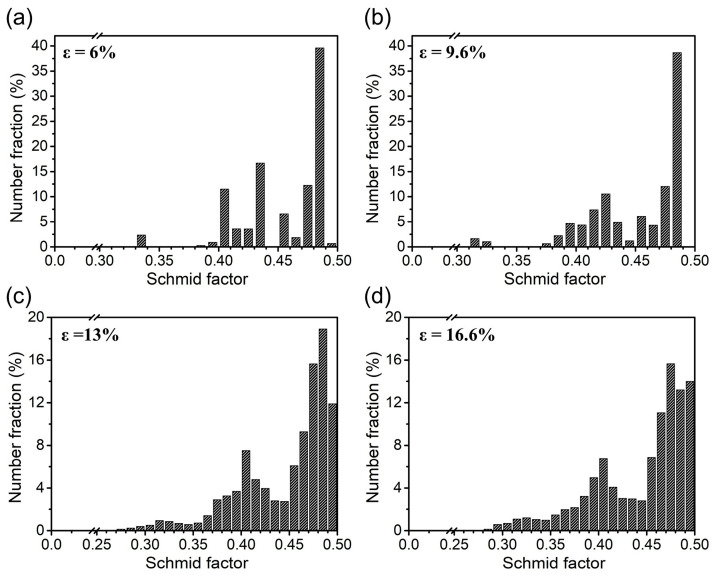
Statistical histograms of Schmid factors at different engineering strain levels. (**a**) ε = 6%; (**b**) ε = 9.6%; (**c**) ε = 13%; (**d**) ε = 16.6%.

Figure 10 illustrates a statistical analysis of the distribution changes of SF at different engineering strain levels. Generally, the grain orientation with an SF larger than 0.4 was defined as soft orientation, while that below 0.4 was defined as hard orientation. At low engineering strain, as shown in Figure 10a,b, SF was mainly distributed above 0.4 and relatively concentrated, indicating that most grains are in a soft orientation, and grains were more likely to initiate dislocation slip and participate in plastic deformation. With the increase of tensile stress and strain, as shown in Figure 10c,d, the density of SF less than 0.4 gradually increased, which represented that more grains were beginning to transform into a hard orientation, resulting in dislocation slip becoming increasingly difficult. In addition, it can be observed that a distribution of SF larger than 0.4 became increasingly dispersed, indicating a significant orientation gradient variation within the grains, and deformation in individual grains became increasingly inhomogeneous.

### 3.5. Evolution of Local Strain

**Figure 11:**
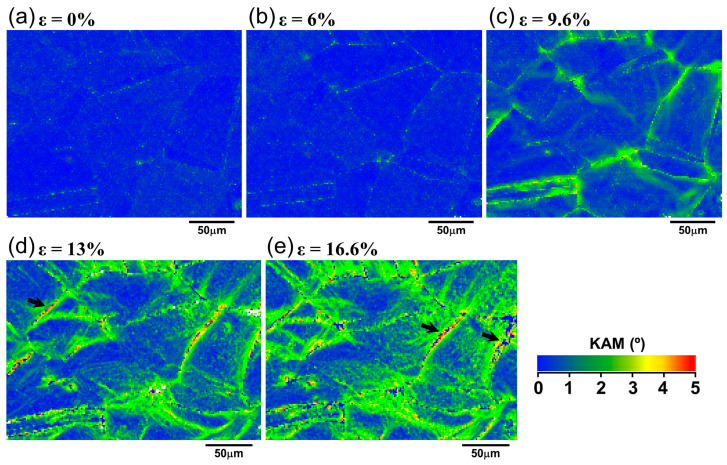
KAM maps at engineering strain of (**a**) 0%, (**b**) 6%, (**c**) 9.6%, (**d**) 13%, and (**e**) 16.6%.

To better understand the local plastic deformation mechanism of IN718 alloy, it is necessary to analyze the variation of local strain with macroscopic strain. KAM is commonly used to qualitatively assess the local strain distribution and dislocation density [25]. Therefore, KAM values can qualitatively evaluate the magnitude of local strain related to the deformation gradient. A low KAM value indicates that the plastic deformation within a grain is homogeneous. On the other hand, a large Kam value represents the non-uniformity of deformation. In general, KAM is less than 1° in recrystallized grains and larger than 1° in deformed grains. Figure 11 shows KAM distribution maps at different macroscopic engineering strains. In the maps, the KAM values were shown in different colors. Blue and red colors represented the lowest and the highest KAM, respectively. It can be observed that the KAMs of some regions increased gradually with deformation, as represented by the increasing fraction of yellow and green colored regions, as shown in Figure 11c–e. This indicates that the local strain gradient is increasing, and the inhomogeneity of plastic deformation is becoming increasingly severe. It is also seen that the color change started from the grain boundary (green in Figure 11c) and gradually expanded into the interior of the grains (Figure 11d,e). It is also clear that, within each grain, the KAM values varied, indicating the inhomogeneity of plastic deformation within individual grains. In addition, red appeared at grain boundaries and TBs at high strain, as indicated by the black arrows in Figure 11d,e. It demonstrates that the KAM in these regions falls in the range of 4–5°, further suggesting the larger deformation inhomogeneity at the grain boundaries.

**Figure 12:**
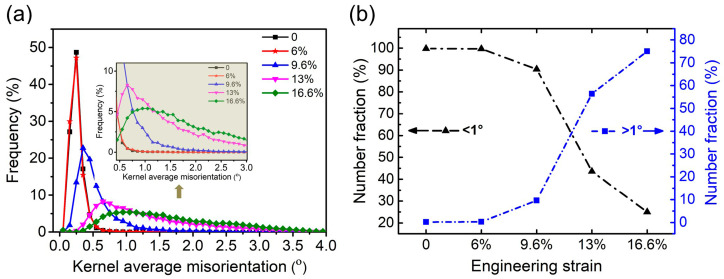
(**a**) Distribution of KAM at different engineering strains; (**b**) statistical chart of different KAM values for the different engineering strains (left—less than 1°, right—larger than 1°).

To quantitatively study the KAM evolution with strain, the distribution of KAM at different strain levels was statistically analyzed, as shown in Figure 12. It can be seen that the distribution of KAM was basically consistent at strains of zero and 6% (black and red curves in Figure 12a), and the KAM values were generally below 0.5°. With the increase in strain, the peaks shifted toward higher KAM values, representing an increased local strain gradient with the increasing strain. The inset in Figure 12a shows that, after the strain of 9.6%, the fraction of high KAM values also increased with the increase in strain, indicating that the degree of local deformation was becoming increasingly inhomogeneous. Figure 12b shows that the statistical variation of KAM values below 1° and above 1° with strain. It is seen that, when the strain was below 9.6%, the local misorientation was mostly less than 1°. As the strain increases, the fraction of the misorientation larger than 1° sharply increases, while misorientations less than 1° rapidly decrease. This indicates that more grains participated in the macroscopic deformation of the sample through the local deformation.

## 4. Discussion

Based on the statistical distribution method, the above results demonstrate the overall changes in the microstructure of the alloy during deformation, and it is found that the evolution of the microstructure exhibits different trends in local areas. Therefore, in the following sections, the deformation behavior of IN718 alloy at the grain scale will be analyzed and discussed using the three adjacent grains (G_1_, G_2_, and G_3_) marked in Figure 5 as the objects.

### 4.1. Coordinated Deformation of Adjacent Grains

#### 4.1.1. Slip System Activity

The SEM micrographs in Figure 4 reveal that dislocation slip plays a crucial role in the tensile deformation process of the alloy at 650 °C. Zhang et al. [26] pointed out that the essence of dislocation slip is the slip motion of dislocations along a specific crystal plane and direction, and the activation of slip systems is closely related to the initial orientation of grains. According to the Schmid law, theoretically, grains with high SF values are in a soft orientation, and the slip system would first reach the critical resolved shear stress under loading, activating the slip of the dislocations to begin deformation. Grains with small SF values are in a hard orientation and are difficult to activate dislocation slip to participate in plastic deformation [27]. However, at a strain of 6%, slip lines were only observed in grain G_1_ (Figure 4c), although the maximum SF value of the slip system in both grains G_1_ and G_2_ was 0.49 (Table 1). This indicates that, under the same load, due to different grain orientations, the shear stress on the slip plane is also not equal. Therefore, even if slip systems in different grains have the same SF, they may not be simultaneously activated to initiate dislocation slip. Gao et al. [17] also found this phenomenon in the tensile testing of IN718 alloy at RT. Due to the two sets of slip systems with high SF values (0.49 and 0.48) in grain G_1_, when the strain increased to 9.6%, another set of slip lines appeared inside grain G_1_, leading to multiple slip deformations. Meanwhile, the slip lines in grain G_2_ were also clearly visible (Figure 9). On the other hand, for grain G_3_, the maximum SF value was 0.4. Compared with grains G_1_ and G_2_, it had a harder orientation and required greater tensile stress to initiate dislocation slip. However, due to the preferential slip deformation of grain G_1_, a large number of dislocations aggregated at the grain boundaries between grain G_1_ and G_3_, forming a strong local stress field (Figure 11c). When the total stress induced by the shear stress of the tensile load, combined with local stress, reached the critical resolved shear stress, dislocation slip in grain G_3_ was initiated, forming clear slip traces on the grain surface. To conclude, during the plastic deformation of polycrystalline materials, the asynchrony of individual grain deformation is not only affected by grain orientation, but also by the stress state between adjacent grains. Meanwhile, the coordinated deformation between the grains also plays a positive role in the plastic deformation.

#### 4.1.2. Grain Rotation

During the tensile deformation, in addition to significant shape changes of individual grains (elongation along the tensile direction), the orientation of the three grains also changed with increasing strain (color changes in Figure 5), indicating that all three grains underwent lattice rotation at a certain angle with deformation. During the tensile deformation of single crystals, the slip direction attempts to rotate towards the direction of the tensile axis [28]. In polycrystalline materials with random orientations, each grain can be regarded as a single crystal, but due to the grain boundaries and interaction between adjacent grains, the rotation behavior of individual grains becomes more complicated. Inverse pole figures (IPFs) can visually demonstrate the evolution of orientation during grain deformation. By observing the position changes of grain orientation in the IPFs, the lattice rotation of each grain can be tracked.

**Figure 13:**
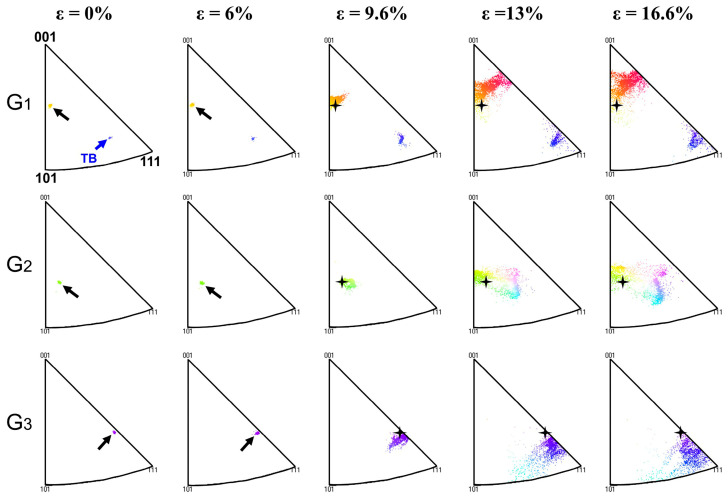
Inverse pole figures in IPF colors displaying the crystallographic orientations of G_1_, G_2_, and G_3_ parallel to the tensile direction at different engineering strains.

Figure 13 shows the evolution of orientation distribution parallel to the tensile direction in grains G_1_, G_2_, and G_3_ with increasing strain. The orientation of the grains in the IPF diagram was represented by the distribution of the IPF data points. In order to better compare the orientation changes before and after deformation, the position of the initial orientation in the IPFs was marked with a black cross star at high strain. At strains of zero and 6%, as indicated by the black arrows, the IPF data points of the three grains showed a dense distribution, indicating that the orientation of the grains is homogeneous. As the strain continued to increase, the data points in grains G_1_, G_2_, and G_3_ became more dispersed and spread in different directions away from their initial positions, which indicates that the grains have rotated, and the lattice rotation inside the grains is not uniform.

**Figure 14:**
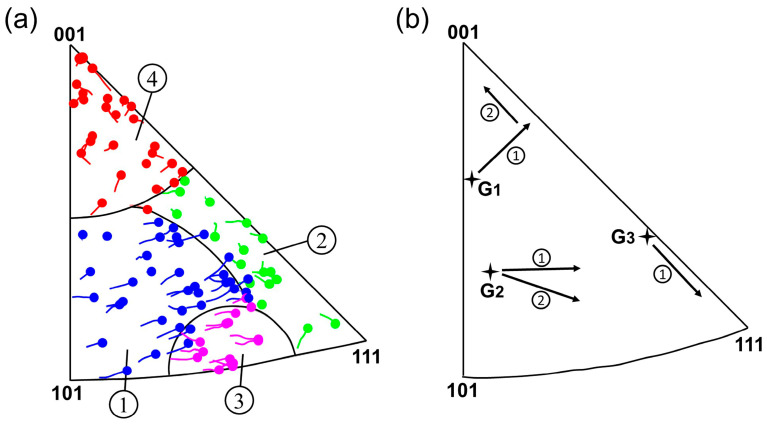
(**a**) Stereographic triangle showing four different grain rotation behaviors of polycrystal aluminum during tension, the symbols mark the final orientation of the tensile direction [31]; (**b**) schematic diagram of rotation paths of grains G_1_, G_2_, and G_3_.

Wu et al. [29] and Poulsen et al. [30] have demonstrated that the rotation behavior of grains is closely related to the initial grain orientation. Winther et al. [31] used in situ XRD technology to study the rotation behavior of a large number of grains in polycrystalline aluminum during tensile deformation. Based on experimental results and lattice rotation theory, the orientation triangle was divided into four regions with different rotation behaviors, as shown in Figure 14a. The initial orientation of a grain determines the region to which the grain belongs. It is seen from Figure 14b that grains G_1_, G_2_, and G_3_ are located in regions 4, 1, and 2, respectively. Therefore, grain G_1_ turns to the [001] pole, grain G_2_ turns to the line connecting the [001] and [111] poles, and grain G_3_ directly turns to the [111] pole along the line connecting the [001] and [111] poles, which basically conform to the rotation path summarized by Winther. However, according to the evolution process of grain orientation represented in Figure 13, the three grains still exhibited significantly different rotation behaviors during deformation, as summarized in Figure 14b. The rotation of grain G_1_ is divided into two steps. First, it rotates in the direction of the line connecting the [001] and [111] poles, and then continues to rotate in the direction of the [001] pole. Unlike grain G_1_, the rotation path of grain G_2_ is more dispersed and mainly divided into two directions, and the rotation in both directions occurs simultaneously. The main reason for this is that the annealing twin inside grain G_2_ greatly increases the difficulty of overall deformation of the grain, making the rotation of grain G_2_ more inhomogeneous (rotation direction and rotation rate). Due to the orientation of grain G_3_ being located near the stable orientation [112], it mainly turns directly to the [111] pole along the line connecting the [001] and [111] poles.

Compared with grains G_1_ and G_3_, the orientation changes inside grain G_2_ are more complex. Figure 6d shows that complete LAGBs were formed inside grain G_2_ at a strain of 13%. However, only scattered short-segment LAGBs appeared near the grain boundaries of G_1_ and G_3_. This is consistent with the orientation changes within the three grains. As shown in Figure 5, the color of grain G_1_ changed from beige to red, and grain G_3_ changed from purple to blue. But, two different colors appeared inside grain G_2_. This indicates the formation of sub-grains composed of new grain boundaries. We speculate that the annealing twin plays a promoting role in the formation of new grain boundaries within grain G_2_. Due to the difficulty of annealing twin deformation, the twin hindered the continuity of lattice gradient changes within grain G_2_. This contributes to the formation of new grain boundaries.

Based on the above analysis, it can be concluded that the initial orientation is the main factor affecting the rotation behavior of grains during the deformation of alloys, while the interaction between grains is the second key factor related to grain orientation. This is consistent with the conclusion reported by Winther et al. [31]. In addition, it can also be found that due to the different initial orientation of grains, the lattice rotation inside individual grains is also non-uniform, which is not only related to the activation of different slip systems inside the grain [32] but also closely related to the different microstructures of grains, such as annealing twins within the grain. The lattice rotation provides another mechanism, in addition to the conventionally multiple slip system mechanism [33], to accommodate plastic deformation within polycrystalline materials.

### 4.2. Transformation Mechanism of Grain Boundaries

The grain boundary distribution map (Figure 6) and statistical histogram (Figure 8) show that, at a strain of 9.6%, a few LAGBs first form in the vicinity of the HAGBs. As the strain increases, the relative fraction of LAGBs significantly increases and gradually expands from the HAGBs to the interior of the grain, while a continuous decrease was observed in HAGBs and TBs, especially for TBs, showing a significant decrease. It should be noted that Figure 8 shows the relative content of different grain boundaries, so the decrease in the HAGBs fraction does not indicate the disappearance or transformation of HAGBs into LAGBs during deformation. This can be confirmed from the grain boundary distribution map (Figure 6), where the HAGBs represented by the blue line did not show a significant decrease or disappearance at high strain. However, the integrity of TBs is partially lost at a strain of 16.6%, which can be seen in Figure 7 by those parts of red-colored TBs that are transformed into black-colored HAGBs. Therefore, in the following sections, the transformation mechanism of the grain boundaries will be discussed from the formation of LAGBs and the loss of TBs.

#### 4.2.1. Formation Mechanism of LAGBs

**Figure 15:**
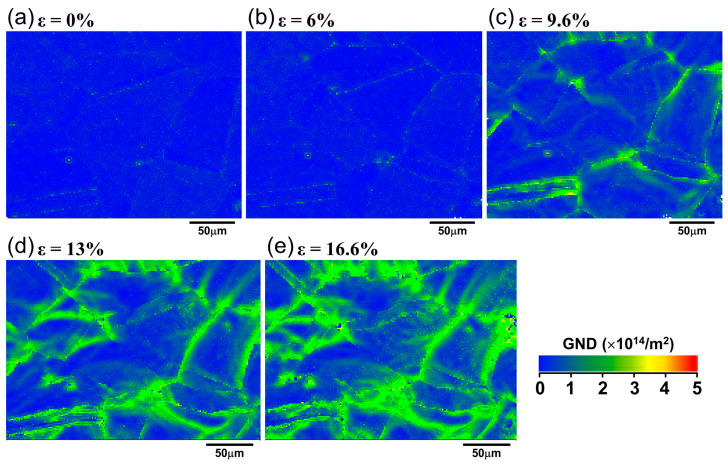
Distribution maps of geometrically necessary dislocations at different engineering strains.

The formation of LAGBs is generally considered a direct result of dislocation movement [29,34]. Figure 15 shows the distribution maps of geometrically necessary dislocations (GND) under different engineering strains. It can be seen that the dislocations first accumulated near the grain boundaries (Figure 15c). Then, the dislocations gradually glided toward the interior of the grains with an increasing strain. This is consistent with the results reported by Mishra et al. [35] and Calcagnotto et al. [36]. During plastic deformation, the dislocation movement was hindered at the grain boundaries and led to dislocation pile-up. With the increase in strain, more dislocations accumulated near the grain boundaries, forming local stress concentrations. Dislocation arrangements along the grain boundaries occurred under the driving of local stress [37]. In order to reduce the deformation storage energy generated during high strain, the dislocations would be arranged in a more ordered state, forming dislocation walls with smaller misorientations. With deformation proceeding, dislocations continued to gather near the dislocation walls, forming more and new dislocation cells or stacking faults, ultimately generating a large number of short-segment LAGBs (shown by the red line in Figure 6d). In addition, due to the uneven rotation of grains, the misorientation of LAGBs gradually increased with the increase of strain and then transformed into deformation-induced grain boundaries with a higher angle (shown by the green line in Figure 6e), subdividing the grains into substructures with different misorientations [34]. Kuo et al. [38] also found that the formation of substructures within the grains is caused by dislocation arrangement in the deformation of pure copper. It is worth noting that the formation of LAGBs mainly begins at high-angle grain boundaries with a strain concentration and gradually expands into the interior of the grains. This is consistent with the results observed by Bibhanshu et al. [39] during the in situ tensile deformation of FeCrAl alloy.

#### 4.2.2. The Loss of TB Integrity

LAGBs provide information on the evolving substructure inside individual grains, and HAGBs give information about changes in the grain structure, such as the formation of deformation twins and the loss of integrity of annealing twins. In the present study, the loss of integrity of annealing twin boundaries was clearly observed at a strain of 16.6% (indicated by purple arrows in Figure 7b), but the formation of deformation twins was not observed. Compared with the matrix grains, annealing twins are more difficult to deform. As evidenced by Figure 5, the orientation of annealing twins inside grain G_1_ and G_2_ remained basically unchanged throughout the deformation process. Therefore, when the matrix grains undergo slip deformation, dislocations are prone to accumulate at the twin boundaries, causing severe strain concentration. According to research of Wang et al. [40], the strain concentration makes the coincidence lattice of the twin boundary to mismatch, which leads to transformation of the TBs into the random high-angle grain boundaries. This is why the peak of the misorientation at 60° in Figure 8a decreased and broadened with increasing strain. In addition, the segmented transformation of TBs into HAGBs was observed in Figure 7b, which further illustrates the non-uniformity of deformation within the grains.

In summary, it can be clearly seen that the deformation ability of each grain is closely related to its initial orientation. The microstructures with specific textures exhibit different deformation characteristics. During plastic deformation, the initial orientation of the grains not only affects the activation of slip systems but also is a key factor affecting the rotation behavior of grains. The formation of LAGBs and the loss of TB integrity provide more specific experimental evidence for the deformation mechanism of alloys at the grain scale. Although these findings were obtained for deformation under uniaxial loading, they establish a dynamic correlation between microstructure evolution and performance, providing important experimental references for clarifying the deformation mechanism of alloys under more complex service conditions. It is worth noting that these findings also apply to the deformation of other polycrystalline alloy materials.

## 5. Conclusions

In this study, the tensile test of IN718 alloy has been conducted at 650 °C using an in situ EBSD tensile stage. Based on advanced EBSD techniques, the microstructural evolution, strain-induced misorientation, grain boundary evolution, activated slip systems, and grain rotation were investigated systematically. The main conclusions are summarized as follows:

(1) The plastic deformation of the IN718 alloy at 650 °C is mainly characterized by the dislocation slip of soft-orientation grains, accompanied by an inhomogeneous lattice rotation within hard-orientation grains. The changes in local misorientation start from the grain boundaries and gradually expand into the interior of grains. The intersection of slip bands and lattice rotation within the grains leads to changes in grain shape;
(2) The initial orientation of the grains has a significant impact on the activation of slip systems. The SF value is not a necessary condition for the activation of slip systems, as it is closely related to the stress state around the grains;
(3) The initial orientation of the grains is the main factor affecting the rotation behavior of grains during the deformation of alloys, while the interaction between grains is the second key factor related to grain orientation. The lattice rotation inside individual grains is non-uniform, which is not only related to the activation of different slip systems inside the grain, but also closely related to the different microstructures of the grains, such as annealing twins within the grain;
(4) The short-segment LAGBs are first formed near the HAGBs with a strain concentration and gradually expand into the interior of the grains. As the density of the LAGBs increases, the LAGBs gradually transform into deformation-induced grain boundaries with higher angles, subdividing the grains into substructures with different misorientations. No deformation twins are observed. Annealing TBs lose their integrity due to the accumulation of dislocations at and in the vicinity of the boundaries at high strain levels.

## Figures and Tables

**Table 1:** Chemical compositions of Inconel 718 alloy studied in this work.

Element	Fe	Cr	C	Nb	Mo	Al	Ti	Co	Mn	V	Ni
wt.%	19.3	17.3	0.07	5.26	3.52	0.52	0.83	0.01	0.05	0.01	Bal.

**Table 2:** Activated slip systems and Schmid factors in grain G_1_, G_2_, and G_3_.

Grain	Slip Trace	Slip Plane	Slip Direction	Schmid Factor
G_1_	S_1_	($\bar{1}$11)	**[0** $\bar{1}$ **1]**	**0.49**
			[101]	0.2
			[110]	0.3
	S_2_	(1$\bar{1}$1)	**[011]**	**0.48**
			$[\bar{1}$01]	0.15
			[110]	0.33
G_2_	S_1_	(1$\bar{1}$1)	[011]	0.2
			$[\bar{1}$01]	0.29
			**[110]**	**0.49**
G_3_	S_1_	(1$\bar{1}$1)	[011]	0.12
			[$\bar{1}$01]	0.27
			**[110]**	**0.39**
	S_2_	(111)	**[0** $\bar{1}$ **1]**	**0.4**
			[$\bar{1}$01]	0.28
			[$\bar{1}$10]	0.12

## Data Availability

The data presented in this study are available on request from the corresponding author. The data are not publicly available due to privacy.
